# Lesions of the Nerve Trunks in Maxillo-Facial Gunshot Wounds

**Published:** 1919

**Authors:** 


***Lesions of the Nerve Trunks in Maxillo-Facial Gunshot Wounds.*** By MM. Leon Imbert and P. Real. *La Restauration Maxillo-Faciale,* March, 1918.

Several important nerve trunks exist in the vicinity of the face and are often injured in maxillo-facial wounds. The concomitant sensory and motary disturbances are of great importance in determining the extent and severity of the lesion, and the authors consider it of interest to summarize the different complications, due to nerve lesions, with which they have had the opportunity of dealing.

Lesions Occurring Most Frequently.

A. *Inferior Maxillary Nerve and its Branches.*

1. Lesions of the *inferior dental nerve.* The section of this nerve is the necessary consequence of a comminutive fracture of the horizontal branch of the mandible. Its section impels a complete disparition of the sensibility in all teeth of the hemimandible situated in front of the fracture, in the bones surrounding the focus of fracture, and in a certain region of the chin.

In several cases of long standing a marked return of sensibility has been observed in the chin, but never did a similar return occur in the teeth. This does not impel any necessary harm for the corresponding teeth. A tooth, even deprived of its innervation, still receives its normal blood and nutrient supply, and therefore its vitality is not specially jeopardized.

2. Lesions of the *lingual nerve* are very often associated with lesions of the inferior dental nerve. The sensibility of the two anterior thirds of the tongue depends from the lingual nerve; its section impels a sensitive and sensory anesthesia of this region.

3. Lesion of the *masseteric nerve.* This nerve is easily injured in fractures of the ascending branch, and this impels the paralysis of the masseteric muscle.

4. Lesions of the *auriculo-temporal nerve.* The corresponding disturbances are : complete insensibility in the parotidian and temporal regions, and in the anterior part of the ear's pavilion. This nerve also regulates the parotid gland's secretion, which explains why the section of this nerve is advisable in cases of salivary fistulae.

5. Lesions of the *trunk of the inferior maxillary nerve.* These are only rarely met with. The authors observed a single case of section of the nerve trunk at the basis of the skull. The symptoms presented by the patient were characteristic symptoms corresponding to the lesions of the inferior dental, lingual, and auriculo-temporal nerves. But next to these, there was a marked unilateral paralysis of the masticatory muscles, and of the masseter and temporal muscles. This can only take place if the section of the nerve trunk is located very near the oval foramen, since the motary fibres innervating the masseter and temporal leave the nerve trunk just where it comes off the skull.

B. *Superior Maxillary Nerve.*

Severe lesions of this nerve trunk are rare, while injuries to the collateral or terminal branches of the nerve are frequent. For instance, injuries to the suborbital nerve have often been observed, impelling complete loss of sensibility in the skin of the orbital region and in the anterior teeth.

C. *Facial Nerve.* Facial paralysis is most frequently observed in all maxillo-facial wounds. Whether or not the inferior facial alone is severed, or the paralysis complete, in no cases do the conditions seem to have any tendency at all to improve.

D. *Terminal Branch of the Hypoglossal.* If this nerve is injured, the extremity of the tongue is deviated towards the injured side and the hemitongue markedly contracted.

### Exceptional Nerve Lesions.

If the tract of the missile runs a certain way along the basis of the skull, the projectile may injure the trunks of the four last pairs of cranial nerves determining a certain number of complex symptoms.

A. *Paralysis of the Glosso-pharyngeal, 9th. Pair.*

The paralysis of the glosso-pharyngeal impels the paralysis of the superior constrictor muscle of the pharynx and the loss of sensibility of the posterior third of the tongue.

1. Paralysis of the superior constrictor of the pharynx. This muscle receives a slight nerve-supply from the vago-spinal, but is mostly innervated by the 9th pair. This muscle's role is to narrow transversally the pharynx while solid food is being swallowed. Its paralysis involves certain functional disturbances; bread and meat can only be swallowed if at the same time the patient drinks a certain quantity of fluid. These symptoms improve generally in course of time, and only a troublesome sensation of too narrow throat remains.

2. Loss of all sensibility in the posterior third of the tongue. This is best proved by using bitter solutions, since sweet and salted sensations are best perceived by the two anterior thirds of the tongue. This research is always difficult, and the results obtained are insufficient to demonstrate the paralysis of the glosso-pharyngeal.

In compound nerves, motor fibers are more easily injured than sensory ones. Only severe injuries of a compound nerve determine sensory disturbances, while motary ones may be caused by a slight compression only. For this reason the paralysis of the superior constrictor presents a great clinical importance for the diagnosis of lesions of the glosso-pharyngeal.

B. *Paralysis of the Pneumogastric, 10th Pair.* This nerve is a sensory one. The diagnosis of lesions of the pneumogastric must be based especially upon the loss of sensibility in the mucous membrane in the pharynx and soft palate. If the section is located below the plexiform ganglion, i. e., after the connection of the pneumogastric and internal branch of the spinal, paralysis of the muscles of the soft palate, of the muscles of the larynx and of the median and inferior constrictors of the pharynx occur concomitantly.

C. *Paralysis of the Spinal, 11th Pair.* The spinal is a motor nerve. A severe lesion of the spinal where it leaves the skull causes the following disturbances:

1. Paralysis of the soft palate. A characteristic deviation of the soft palate appears during the slightest reflex movements, the uvula being attracted towards the sound side. Concomitant alterations of the voice and troubles in the deglutition always accompany a true paralysis of the soft palate.

2. Paralysis of the median and inferior constrictors of the pharynx is generally of slight importance, since it may be compensated for by the styloid and suprahyoid muscles action.

3. Paralysis of the muscles of the larynx. This is easily detected by marked changes in the voice. Electric reactions of the sterno-cleidomastoid and trapezius muscles should always be determined in such cases.

D. *Paralysis of the Hypoglossal, 12th Pair.* The terminal branches of this nerve end in the muscles of tongue, and its descending branch in the hyoid muscles.

E. *Associated Paralysis — Different Clinical Types.* All these different motor and sensory disturbances may be found associated, forming certain syndromes of great clinical importance. Five principal types have been studied :

1. *Avellis Syndrome.* Corresponds to the lesion of the internal branch of the spinal; its features are the paralysis of the soft palate and larynx. Occurs very seldom (twice out of 44 cases).

2. *Schmidt Syndrome.* Equally rare — due to alterations of all the fibers of the spinal. Corresponding disturbances are both motary and sensory.

3. *Jackson's Syndrome.* Due to injuries of both the hypoglossal and spinal.

4. *Vernet's Syndrome.* Due to concomitant lesions of the 9th, 10th and 11th nerves.

*Total Syndrome of the 4 last Cranial Nerves.*

5. *Tapia's Syndrome.* Lesions of the vago-spinal and 12th nerve.

The syndromes, numbers 3, 4 and 5, are necessarily due to a lesion located just beneath the basis of the skull; this presents some importance for localizing a projectile prior to its removal.

